# Harnessing the Power of Fermented Tea to Improve Gut Microbiota and Combat Obesity Epidemic

**DOI:** 10.3390/biology13100779

**Published:** 2024-09-28

**Authors:** Ruyi Zhang, Qiling Li, Yuxuan Gu, Wenli Liao

**Affiliations:** 1 School of Pharmacy, Hubei University of Chinese Medicine, Wuhan 430065, China; zhangry@hbust.edu.cn; 2 Basic Medical School, Hubei University of Science and Technology, Xianning 437100, China; 3 School of Basic Medical Sciences, Hubei University of Chinese Medicine, Wuhan 430065, China

**Keywords:** fermented tea, obesity, gut microbiota, metabolic health, polyphenols

## Abstract

**Simple Summary:**

This research examines the efficacy of fermented tea in promoting gut health and addressing obesity. The human gastrointestinal tract contains a diverse array of bacteria that affect metabolism and health outcomes. Obesity poses a significant public health concern, often arising from an imbalance in gut microbiota. Fermented tea contains beneficial compounds, notably polyphenols. These compounds can modify the gut microbiota composition, which may assist in managing weight and enhancing metabolic health. The study aims to clarify how fermented tea influences obesity through gut bacteria alterations. The results reveal that fermented tea fosters the proliferation of beneficial bacteria while inhibiting harmful strains, potentially improving energy balance and mitigating obesity. Such findings could inform effective dietary interventions to tackle obesity and associated health challenges, ultimately benefiting public health and reducing healthcare expenditures.

**Abstract:**

The global rise in obesity rates has prompted a thorough evaluation of dietary strategies that may alleviate this metabolic issue. Fermented tea, a beverage rich in polyphenols and catechins, has emerged as a viable therapeutic option for obesity management. This review discusses the role of fermented tea in modulating the gut microbiome, a critical factor in energy regulation and obesity. We explore how the bioactive components in fermented tea influence gut health and their implications for metabolic health. Fermented tea may inhibit weight gain and fat accumulation in obese animal models, likely by promoting beneficial bacteria and suppressing harmful species. Changes in the production of short-chain fatty acids and improvements in gut barrier integrity are linked to enhanced insulin sensitivity and reduced inflammatory markers, essential for effective obesity management. However, barriers remain in applying these findings in clinical settings, such as the need for standardized fermentation techniques and accurate dosage assessments. This review underscores the therapeutic potential of fermented tea in obesity treatment and advocates for further research to enhance its integration with public health initiatives.

## 1. Introduction

Obesity has surfaced as a global public health crisis [1]. The World Health Organization (WHO) indicates that over 4 billion individuals are classified as overweight, with more than 1.5 billion struggling with obesity, which is double the rate since 1990 [2,3,4]. This condition ties to various health complications, including cardiovascular diseases, type 2 diabetes, and specific cancers, ultimately raising morbidity and mortality rates [5,6,7]. The rising rates of obesity prompt the need for new and effective management strategies.

Tea stands as one of the globe’s most widely consumed beverages [8]. It holds value not only for its cultural relevance but also for its potential health benefits [9,10,11]. Recently, fermented tea has attracted interest regarding its possible impact on obesity management [12,13,14]. This type of tea undergoes a microbial fermentation process, which modifies the leaves’ chemical composition [15,16]. As a result, the beverage becomes rich in bioactive compounds like polyphenols, catechins, theaflavins, caffeine, and thearubigins. Previous research verifies that these bioactive compounds can aid in managing obesity effectively [14,17,18,19].

The gut microbiome, a diverse consortium of microorganisms inhabiting the digestive system, plays a crucial role in human health and illness, particularly in obesity [20,21,22]. Dysbiosis in the gut microbiome, marked by an excess of Firmicutes and a shortage of Bacteroidetes, is associated with obesity [23,24,25]. Increasing evidence indicates that fermented tea may affect the makeup and activity of the gut microbiome. By altering this microbiome, it presents an innovative strategy for managing obesity [26,27,28,29].

In this review, we investigate the complex role of fermented tea in combating the global obesity epidemic. We analyze the bioactive compounds found in these beverages and their effects, aiming to clarify their potential for obesity management. Additionally, we examine the nuanced interaction between fermented tea and gut microbiota, assessing how these dynamics might foster weight loss and enhance metabolic health. Our review includes an evaluation of existing clinical studies, highlighting the potential of fermented tea in obesity treatment while also recognizing the ongoing challenges in clinical settings. To compile this foundational overview, we systematically reviewed the literature from credible sources like PubMed, Google Scholar, the Web of Science, and CNKI, concentrating on terms such as “fermented tea”, “obesity”, and “gut microbiome”. This integrative methodology enables us to provide a comprehensive view of the historical, cultural, and scientific relevance of tea, as well as its market developments over the past forty years, creating a solid framework for understanding the role of fermented tea in the ongoing fight against obesity.

## 2. Evaluation of Bioactive Compounds in Fermented Tea for Obesity Treatment

Fermented tea stands as a beverage steeped in tradition and nutritional significance [30]. Its potential role in obesity management has recently attracted considerable interest [31,32]. This beverage is categorized into four main types: lightly fermented, semi-fermented, fully fermented, and post-fermented, as illustrated in Table 1 [8]. Prominent examples like kombucha and brick tea are examined in detail in Table 2 [8,15,30]. The essential bioactive components of fermented tea encompass tea polyphenols, catechins, theaflavins, caffeine, and thearubigins. These compounds demonstrate beneficial impacts on human health, aiding in weight regulation and obesity mitigation [17,33,34,35,36].

Tea polyphenols and catechins regulate lipid metabolism [17,37]. They reduce the triglyceride (TG), total cholesterol (TC), and low-density lipoprotein cholesterol (LDL-C) levels in the blood. Additionally, they lower the lipid content in other organs and tissues, such as the liver and kidneys [38,39]. This action helps prevent obesity and hyperlipidemia while decreasing the incidence and mortality of cardiovascular diseases like atherosclerosis and coronary heart disease [40,41,42]. Currently, the mechanisms of tea polyphenols and catechins for weight loss and lipid reduction include several pathways: First, they inhibit enzymes that are associated with fat synthesis. Green tea catechins reduce the expression and activity of various fat synthesis-related enzymes and modulate the activity of cholesterol synthesis enzymes [17]. Second, they promote fatty acid oxidation, which increases energy expenditure. Previous research shows that epigallocatechin gallate enhances the activity of adrenaline and regulates β-oxidation in the liver and skeletal muscle, leading to fat loss and improved energy metabolism [43]. Moreover, catechins can suppress one’s appetite. They enhance fatty acid oxidation in the liver, possibly regulating appetite. Previous studies demonstrated that epigallocatechin gallate reduced appetite by 50% to 60% in experimental rats [44]. However, its effects on appetite suppression in human trials are limited. Lastly, they inhibit nutrient absorption. In vitro experiments suggest that catechins inhibit digestive enzymes in the gastrointestinal tract and form complexes with specific transporters on the brush border of intestinal cells [44,45]. This action decreases the absorption and utilization of glucose and lipids. Epigallocatechin gallate also reduces the solubility and size of lipid substances, interfering with normal micelle formation, leading to inhibited activities of gastric and intestinal lipases, and, consequently, suppressing lipid breakdown [46,47,48,49,50].

Caffeine represents another crucial bioactive element in fermented tea [11,35]. It promotes fat oxidation by elevating metabolic rates and aiding the mobilization of fatty acids, which increases energy expenditure [18]. Caffeine inhibits adenosine receptors, leading to an elevated release of neurotransmitters that boosts metabolic rates and fatty acid breakdown. Furthermore, caffeine activates the central nervous system, improving alertness and focus, thereby indirectly influencing energy expenditure [33].

Theaflavins and thearubigins stand out as distinctive compounds generated during fermentation [46,51]. They enhance lipid metabolism by promoting fat oxidation and reducing blood lipid concentrations. These compounds may also affect the host’s metabolic conditions by altering the gut microbiota’s composition and function. Studies show that theaflavins and thearubigins can influence gut microbiota, leading to an increase in beneficial species like bifidobacteria and lactobacilli [51]. These advantageous microbes play a crucial role in improving gut health, bolstering gut barrier integrity, alleviating inflammation, and potentially influencing fat storage and metabolism [38,52,53].

Fermented tea polysaccharides can regulate obesity [54]. First, they promote the growth of beneficial microbiota, such as lactobacilli and bifidobacteria, while inhibiting harmful bacteria, thereby improving the structure and function of the gut microbiome. This adjustment enhances the gut barrier function and reduces intestinal inflammation, subsequently inhibiting obesity progression [54,55]. Additionally, fermented tea polysaccharides stimulate the metabolic activities of beneficial gut bacteria, increasing the production of short-chain fatty acids. These fatty acids play crucial roles in regulating energy intake, enhancing satiety, and improving insulin sensitivity. Furthermore, fermented tea polysaccharides have shown the ability to lower the levels of inflammatory factors associated with obesity. They achieve this by regulating the gut mucosal barrier function and suppressing inflammatory signaling pathways to diminish intestinal inflammatory responses [56].

## 3. Research on the Correlation between Gut Microbiota and Obesity

The global obesity epidemic poses an undeniable crisis [1]. The World Health Organization reports that over 4 billion people worldwide are overweight, with more than 1.5 billion suffering from obesity [57]. This issue is not confined to developed nations; it is rapidly escalating in many developing countries as well. The data indicate that global obesity rates have doubled since 1990, posing a significant challenge to public health management [2,58]. The sharp rise in obesity is closely associated with various factors. Socio-economic development has led to lifestyle changes, including dietary shifts, decreased physical activity, and environmental changes, all of which are significant contributors to the obesity epidemic [5]. Genetics, metabolic disorders, hormone levels, and intestinal microbiota also play crucial roles in the development of obesity [1,23,25,59,60]. As the obesity epidemic continues to spread, innovative strategies for treating and managing obesity are urgently needed. Recent studies suggest that modulating the gut microbial community could be a potential new approach to obesity treatment [61,62]. The intestinal microbial community is integral to physiological functions such as digestion, nutrient absorption, and metabolic regulation, and it is closely linked to obesity [63,64,65]. Consequently, prebiotic, and probiotic products, including fermented teas, have garnered significant attention as novel methods for modulating the gut microbial community in obesity treatment [66,67]. Given the escalating obesity problem, research into new therapeutic strategies is especially urgent. The global obesity epidemic underscores the necessity of addressing a combination of factors and exploring more precise and individualized interventions to tackle this global public health challenge.

Gut microbes form microbial communities with significant physiological functions within the human body, and their connection to obesity is profound [68]. Previous research indicates that the gut microbial composition in obese individuals is markedly differs from that of normal-weight individuals, with variations in the abundance, diversity, and population structure. Firmicutes and Bacteroidetes are two major groups of intestinal microorganisms that are pivotal in the progression of obesity [25]. An increase in the abundance of Firmicutes, which are bacteria capable of efficiently utilizing dietary energy, leads to heightened energy intake, which, in turn, fosters weight gain and the advancement of obesity. Conversely, Bacteroidetes primarily contribute to fiber breakdown, facilitating digestion and absorption and maintaining intestinal flora balance. Obese individuals often exhibit a relative surplus of Firmicutes and a relative deficit of Bacteroidetes, indicating that an imbalance in gut microbes may be associated with the development of obesity [25]. Gut microbes can affect various aspects of energy metabolism, hormone secretion, and immune regulation in the host, thereby influencing the onset and progression of obesity [69,70]. By adjusting the composition and function of intestinal microorganisms, obesity symptoms can be effectively combated, offering a novel therapeutic strategy. Fermented tea, as a natural prebiotic food, may aid in weight control and obesity treatment by modulating gut microbes, decreasing the proportion of Firmicutes, and increasing the proportion of Bacteroidetes [71,72]. The connection between intestinal microorganisms and obesity is tight, and an imbalance in intestinal microorganisms may be a key factor in the development of obesity. Regulating the composition and function of gut microorganisms could pave the way for new approaches and strategies in obesity treatment and offer new hope for disease prevention and control.

## 4. The Relationship between Fermented Tea and Intestinal Flora in Obesity Treatment

Regulation of intestinal flora: Regulating the structure of intestinal flora is crucial in treating obesity. Recent research increasingly suggests that altering the intestinal microbial composition can significantly reduce the obesity risk and mitigate symptoms [25]. Fermented tea, a traditional beverage, shows promise in this modulation. Probiotics and beneficial microorganisms, such as lactobacilli and bifidobacteria, are plentiful in fermented tea and play a key role in managing the intestinal flora structure [73]. These probiotics help maintain a balanced gut microbiome by competitively inhibiting harmful bacteria and supporting the growth of beneficial species. Additionally, fermented tea is rich in natural cellulose and polyphenols, which not only provide nourishment for good bacteria but also influence metabolic processes that are related to obesity by regulating intestinal flora metabolism [74,75]. Therefore, fermented tea presents a valuable approach for managing intestinal microbiota in obesity treatment. Future research should aim to investigate the specific regulatory mechanisms of fermented tea on the gut microbiota in obese patients, providing further theoretical insights for developing innovative obesity treatment strategies.

Promoting short-chain fatty acids: Promoting short-chain fatty acid production is a key microbial regulatory strategy in the gut, being crucial for treating obesity with fermented tea [76,77,78]. Studies reveal that short-chain fatty acids perform various physiological functions in the intestinal tract, including regulating intestinal mucosal cell growth, maintaining intestinal mucosal barrier integrity, and suppressing inflammatory responses [70]. By enhancing short-chain fatty acid production, the metabolic status of obese patients can be improved, the body mass index reduced, and the blood sugar and lipid levels optimized [78]. Intestinal probiotics, including lactobacilli and bifidobacteria, are the primary source of short-chain fatty acids, which are produced through their fermentation metabolites [79]. Therefore, increasing one’s probiotic intake can effectively boost intestinal short-chain fatty acid production. Moreover, certain studies indicate that active ingredients in fermented tea, including tea polyphenols and catechins, can significantly promote intestinal probiotic growth and metabolic activity, thereby increasing short-chain fatty acid production [74,80]. Beyond probiotics, intestinal microbial diversity also significantly contributes to short-chain fatty acid production. Obesity often impairs probiotic abundance and diversity, leading to reduced short-chain fatty acid production [54,76]. Therefore, by adjusting the intestinal microbial community structure and enhancing probiotic diversity, the short-chain fatty acid content can be effectively increased, playing a therapeutic role in obesity treatment. Overall, promoting short-chain fatty acid production is an important strategy for regulating gut microbes in obesity treatment through fermented tea. By enhancing probiotic absorption and optimizing the intestinal microbial community structure, the short-chain fatty acid content can be effectively increased, improving the metabolic status of obese patients. This provides an important theoretical basis and practical guidance for exploring new strategies to treat obesity.

Anti-inflammatory: The intestinal inflammatory response is a common complication in obese patients and is crucial for overall obesity treatment [81]. Studies have shown that fermented tea can effectively inhibit the onset and development of intestinal inflammatory responses by modulating gut microbes [44]. Components rich in medicinal properties, such as tea polyphenols and catechins in fermented tea, have been shown to play a significant role in reducing inflammatory cytokine levels, modulating the intestinal mucosal barrier function, and inhibiting inflammatory signaling pathways [70,82,83]. Further research has found that fermented tea optimizes the intestinal microecological environment by increasing the probiotic community and reducing harmful bacteria, significantly reducing intestinal inflammatory responses [84,85,86]. Additionally, beneficial active ingredients in fermented tea exhibit obesity-related immunomodulatory effects, inhibit inflammatory cell-mediated immune responses, and reduce obesity-induced intestinal inflammation [70]. Overall, as a potential strategy for treating obesity and preventing intestinal inflammatory responses, fermented tea is expected to offer an effective and natural method to inhibit intestinal inflammatory responses in obese patients by modulating the composition and function of intestinal microorganisms. This strategy not only holds potential clinical applications but also provides new insights and approaches for comprehensive obesity treatment and the management of associated complications.

Improving intestinal barrier function: Maintaining and improving the intestinal barrier function is essential for intestinal health and disease prevention [87]. Studies indicate that active ingredients in fermented teas can effectively enhance the intestinal barrier function by regulating the composition and metabolic activity of intestinal microorganisms [65,88,89]. These ingredients promote beneficial bacterial proliferation and inhibit harmful bacterial growth. Components such as polyphenols and probiotics in fermented tea have been shown to increase the number and activity of probiotics (e.g., Lactobacillus, Bifidobacterium) in the intestinal tract. These probiotics can regulate the intestinal environment and reduce harmful bacteria in the intestine, thereby mitigating damage to the intestinal lining caused by harmful bacteria and improving the intestinal barrier function [90,91]. The active ingredients in fermented tea can also regulate short-chain fatty acid production in the intestinal tract, increase the metabolic rate of intestinal mucosa cells, and enhance the anti-inflammatory and repair capabilities of the intestinal mucosa [92]. These effects help maintain the integrity and function of the intestinal mucosa and promote the normal function of the intestinal barrier. As a gut microbial modulation strategy for obesity treatment, fermented tea significantly improves intestinal barrier function and maintains intestinal health. This provides a theoretical and experimental basis for the application of fermented tea in obesity treatment and disease prevention.

## 5. Clinical Practice and Prospects of Fermented Tea in Obesity Treatment

### 5.1. Research on the Clinical Application of Fermented Tea in Obesity Treatment

The results from these experiments indicate that fermented tea substantially curtails weight gain and diminishes fat accumulation in obese animals [93]. Furthermore, fermented tea has shown potential in regulating blood sugar levels, contributing to the improved metabolic health of obese animals [94,95]. Regarding the gut microbiome, fermented tea modulates the relative abundance of beneficial bacteria, such as lactobacilli and bifidobacteria, while suppressing the growth of harmful bacteria, thereby enhancing the intestinal health of obese animals [96,97,98,99]. Further animal model experiments suggest that fermented tea can influence intestinal barrier function, inflammation levels, and metabolite production by modulating the gut microbiome, which, in turn, positively affects obesity status [100,101,102,103,104]. Animal experimentation results offer new research perspectives for the modulation of gut microbiota associated with obesity, providing a theoretical foundation for clinical applications.

Based on results from animal studies, fermented tea has recently found applications in clinical interventions [16]. Green tea extract shows limited effects on weight loss and weight maintenance in overweight or obese adults. An analysis of 15 weight loss studies and 3 weight maintenance studies reveals that participants using green tea extract exhibited minimal, statistically insignificant reductions in their weight, body mass index (BMI), and waist circumference compared to the control group. Hence, the efficacy and safety of green tea extract as an adjunct for weight loss and maintenance require further investigation [98,102]. In another clinical study, some evidence suggests that consuming Pu-erh tea for eight weeks can significantly reduce weight and BMI in overweight and obese populations [105]. Currently, clinical data on the effectiveness of fermented teas for treating obesity remains sparse, highlighting the urgent need for extensive clinical research to confirm its benefits.

Fermented tea, a beverage produced through microbial fermentation, raises safety concerns among consumers and researchers alike [106]. The safety of fermented tea primarily hinges on its microbial safety [107]. During fermentation, tea leaves may encounter microbial contamination, notably from molds. Mycotoxins generated by molds, such as aflatoxins, pose significant health risks [108,109]. Recent studies have advanced in detecting mycotoxins in fermented tea. For instance, researchers have employed polyphenol precipitation and dual-column tandem methods to mitigate the interference of secondary metabolites in detecting mycotoxins [110]. They established a confirmatory LC-MS/MS method for aflatoxin detection in fermented tea and completed associated risk assessments [15,111,112]. Acute toxicity testing constitutes an important method for evaluating food safety [67]. Some studies have assessed the safety of artificially inoculated solid-state fermented Dark Tea using acute toxicity tests. Their results indicate that the LD50 (lethal dose for 50% of subjects) for artificially inoculated Dark Tea falls within the practically non-toxic range, suggesting high safety for consumption. Long-term safety of fermented tea also concerns consumers [15]. While active compounds such as tea polyphenols and catechins provide health benefits, excessive consumption may lead to adverse effects like headaches, dry mouth, and palpitations [15,94]. Thus, moderate consumption of fermented tea, complemented by personalized selection of varieties and drinking methods, remains crucial for ensuring long-term safety.

### 5.2. Prospects for the Application of Fermented Tea in Obesity Treatment

Socio-economic benefits analysis is a vital tool for evaluating the social and economic impacts of a policy, project, or technology. In the context of investigating the microbial modulation strategy of fermented tea for treating obesity, it is essential to consider its socio-economic benefits [15]. Utilizing fermented tea to treat obesity can decrease the risk of chronic diseases such as cardiovascular disease and diabetes, thereby alleviating the strain on healthcare systems [8]. Enhancements in population health can lead to decreased absenteeism and productivity loss due to illness, which, in turn, can yield greater economic benefits. The production and distribution of fermented tea as a natural health product can stimulate growth in related industries, contribute to the economic expansion of associated businesses, generate employment opportunities, and potentially exert a positive impact on a country’s overall economy. Furthermore, if the fermented tea treatment program successfully aids in weight reduction for obese patients, it can diminish their reliance on medications and medical services, lower the overall healthcare costs, and promote the efficient use of healthcare resources [113]. By analyzing the socio-economic benefits of the gut microbial modulation strategy for obesity treatment with fermented tea, the therapy’s potential social and economic impacts can be more thoroughly evaluated. This analysis provides a critical reference for policy formulation and decision making.

Marketing fermented tea products presents a complex and challenging endeavor. Initially, consumers exhibit a limited understanding of fermented tea and lack adequate awareness and trust in these products. The prevalence of traditional tea beverages in the market intensifies competition for fermented tea products during promotional efforts [107,114]. Furthermore, effectively communicating the nutritional and health benefits of fermented tea in a scientific and engaging manner is crucial to capturing consumer interest and trust [114]. However, the scarcity of relevant research and data poses certain challenges to marketing initiatives.

Additionally, establishing and expanding market channels is a critical aspect of promoting fermented tea products. The unique production process and shelf-life considerations of fermented tea necessitate advanced technical support and specialized expertise in channel development. Consequently, identifying appropriate sales channels, fostering stable relationships, and ensuring consistent product quality are essential elements of the marketing strategy. The marketing process must also address the challenge of varying consumer preferences and needs. Consumers from different age groups, geographic regions, and cultural backgrounds display significant differences in their acceptance of fermented tea products [114,115]. Therefore, it is imperative to devise targeted marketing strategies and offer personalized services and product customization to cater to the diverse needs of various consumer segments. To successfully navigate the marketing challenges of fermented tea products, it is essential to adopt scientific and rational strategies and methods. This approach should be coupled with marketing and brand-building concepts, as well as a commitment to continuous innovation and enhancement of product quality. These efforts are vital to effectively overcoming market barriers and earning the favor and trust of consumers.

## 6. Discussion

**Figure 1:**
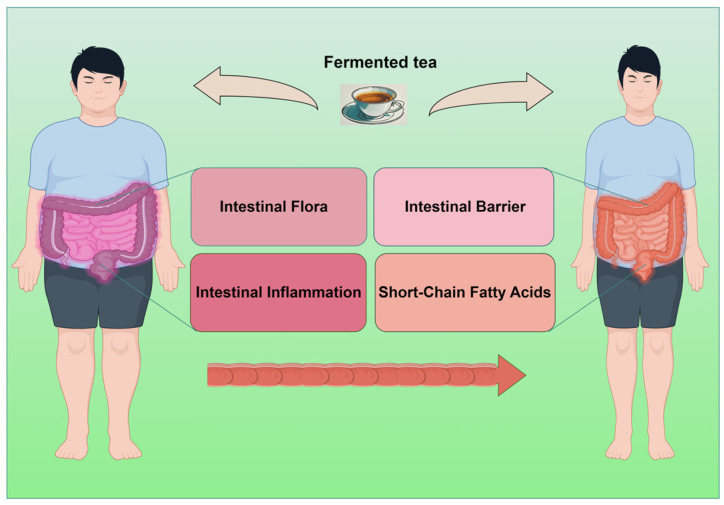
Fermented tea regulates obesity by improving gut microbiota, increasing short-chain fatty acids, reducing inflammation, and enhancing intestinal barrier function.

The worldwide rise in obesity has reached critical levels, necessitating immediate and effective management strategies. This review compiles current research focused on the impact of fermented tea in modulating gut microbiota and combating obesity. It underscores the promise of these traditional beverages as natural therapeutic agents. Fermented tea contains abundant bioactive compounds, like polyphenols and catechins, which have demonstrated the ability to influence the gut microbiome composition, enhance metabolic health, and alleviate obesity-related symptoms in both animal models and human studies, as shown in Figure 1.

Fermented tea holds significant cultural value. As a readily consumable drink, it presents a viable alternative to conventional medicine. Its non-toxic properties and low incidence of severe side effects make it an attractive choice for prolonged use in weight management and enhancing metabolic health. Given its convenience and safety, fermented tea may offer a simple and practical method for weight control and promoting metabolic wellness.

The therapeutic potential of fermented tea holds promises, yet several challenges must be recognized. Cultural variations in preparation and consumption may lead to inconsistent effectiveness in treating obesity. The difficulty in identifying the optimal dosage further complicates its use, as the impact of fermented tea on weight management and metabolic health may vary with dosage. Furthermore, the fermentation process, which is critical for generating bioactive compounds, can be influenced by numerous factors, ultimately affecting product consistency and quality. Future research must focus on standardizing production processes, establishing evidence-based dosage guidelines, and investigating the mechanisms by which fermented tea impacts gut microbiota and metabolic outcomes. Addressing these challenges is essential for incorporating fermented tea into evidence-based obesity treatment approaches.

In conclusion, fermented tea represents a promising strategy for managing obesity by modulating gut microbiota. The bioactive compounds within these teas demonstrate a capacity for supporting weight regulation and improving metabolic health. Clinical evidence underscores the efficacy of fermented tea in curbing weight gain and enhancing metabolic conditions in obese animal models. However, additional research remains crucial to clarify the specific regulatory effects of fermented tea on the gut microbiota in obese individuals and to tackle safety and marketing issues. The findings of this review indicate that fermented tea could serve as a valuable resource in the prevention and treatment of obesity, offering new possibilities for disease management.

## 7. Conclusions

This study highlights the promise of fermented tea as a natural strategy to enhance gut health and address the obesity crisis. It elucidates the complex interplay between fermented tea intake and gut microbiota modulation, suggesting that this beverage could serve as an effective dietary intervention for obesity management. Key components, such as polyphenols and catechins, of fermented tea appear to affect gut microbial diversity, promoting beneficial bacteria while inhibiting harmful strains. This alteration in the microbial composition correlates with enhanced energy metabolism and decreased fat storage, which are crucial for obesity control. The evidence indicates that consistent consumption of fermented tea may lead to notable reductions in body weight and fat levels, alongside improvements in metabolic markers like insulin sensitivity. These encouraging findings indicate a need for further exploration into the specific mechanisms and optimal formulations of fermented tea for clinical applications in obesity treatment. Furthermore, the implications of this research extend beyond individual health, potentially alleviating pressures on healthcare systems and encouraging healthier lifestyles. Future studies must aim to validate these results in larger clinical settings and determine the most effective types and dosages of fermented tea for therapeutic purposes. Overall, this work adds to the increasing evidence supporting fermented tea as a viable method to improve gut health and combat obesity, holding potential for innovative public health strategies to tackle this global issue.

## Figures and Tables

**Table 1:** Fermented teas exhibit varying degrees of fermentation.

Fermented Tea	Characteristics
Lightly-fermented tea	Not fermented, natural fragrance, refreshing taste, and bright green color.
Semi-fermented tea	Fermentation controlled between 20% to 70%, featuring both the fresh fragrance and mellow taste of green tea and the color and fruity aroma of black tea.
Fully fermented tea	100% fermented, resulting in a bright red or dark red color and a unique aroma.
Post-fermented tea	Processed through pile fermentation, offering a rich, smooth taste and known for weight loss and reducing “three highs” (blood pressure, blood sugar, and blood lipids).

**Table 2:** The classification of fermented teas encompasses a variety of types.

Fermented Tea	Characteristics
Red tea (black tea)	Red tea, commonly known as black tea outside of China, is one of the most widely consumed fermented teas. It undergoes a high level of oxidation, resulting in leaves that are typically dark brown to black in color. Notable varieties include Zhengshan Xiaozhong, Qimen Hongcha, and Dianhong.
Black tea (post-fermented tea)	Black tea is a post-fermented tea characterized by a very high degree of oxidation, leading to leaves that are deep black. Key varieties are Pu’er, Liu Bao, and Anhua Black Tea.
Yellow tea:	Yellow tea is a category of tea that falls between green and red teas in terms of the fermentation level, resulting in leaves that display a yellow hue. Representative varieties are Junshan Yinzhen, Mengding Huangya, and Huoshan Huangya.
White tea	White tea is characterized by a low level of fermentation, primarily achieved through natural oxidation. It is known for its delicate processing and light flavor profile. Major varieties include Baihao Yinzhen, Bai Mudan, and Shoumei.
Kombucha	Kombucha is a fermented beverage produced by fermenting tea with a symbiotic culture of bacteria and yeast (SCOBY), resulting in a slightly effervescent, acidic beverage. It typically contains tea, sugar, acetic acid bacteria, and yeasts.
Rice wine tea	Rice wine tea is a unique tea produced by fermenting tea leaves with rice wine, which imparts a distinctive flavor and aroma to the beverage.
Fruit-infused fermented tea	This category of tea is created by fermenting tea leaves in combination with fruit, which imparts additional fruit flavors and aromas to the tea. Common examples include strawberry-infused fermented tea and lemon-infused fermented tea.
Herbal-infused fermented tea	Herbal-infused fermented tea is made by fermenting tea leaves with various herbs, which can enhance the tea’s health benefits and flavor profile. Notable examples are mint-infused fermented tea and ginger-infused fermented tea.
Specialty fermented teas	Specialty fermented teas encompass regionally specific teas such as Tibetan tea and brick tea, which are distinguished by their unique local flavors and traditional production methods.
